# TrackChain: Hyperledger based pharmaceutical supply chain – Resource utilization perspective

**DOI:** 10.1016/j.heliyon.2023.e23250

**Published:** 2023-12-07

**Authors:** C.M Naga Sudha, Jesu Vedha Nayahi J

**Affiliations:** a Department of Computer Technology, Anna University - MIT Campus, Chennai, Tamilnadu, India; b Department of Computer Science and Engineering, Anna University – Regional Campus, Tirunelveli, Tamilnadu, India

**Keywords:** Pharmaceutical drugs, Supply chain, Hyperledger sawtooth, Hyperledger fabric, Resource consumption

## Abstract

A distributed and decentralised ledger system shared by a variety of users in a peer-to-peer network is defined as a blockchain. Through its decentralised approach, it makes pharmaceutical products more accessible and secure for vendors, while they can maintain and access their inventories themselves. Between a company and its suppliers, a supply chain is used to produce and distribute a specific product or service, whose efficiency is increased with its integration in the supply chain. For efficient and transparent tracking of products, blockchain will be the right choice. In view of blockchain being incorporated into the supply chain system, it solves many logistical issues that supply chains face, such as appropriate data access, ensuring data quality, and many more. This can increase the traceability of the material supply chain and improve the maintenance of the list when there are several products to be supplied. Therefore, the concept of supply chain was developed in two platforms namely, Hyperledger Fabric and Hyperledger Sawtooth. Finally, Hyperledger Sawtooth was found to be well suited for creating decentralised apps or platforms with respect to resource utilization It enables developers to separate their application domain from the core system so that business rules for apps can exist without requiring knowledge of the primary platform's underlying architecture. The proposed system shows that Hyperledger Sawtooth framework consumes lower CPU than Fabric which helps in increasing the number of transactions.

## 1 Introduction

Healthcare Supply Chain is considered as a network of independent entities which includes raw materials, producers, distributors, pharmacies, hospitals and patients [1]. Tracking of supplies is not a trivial task as it involves several factors such as competition among stakeholders, inadequate information and centralized control. Thus, complexity results in inefficiencies and aggravate the mitigating task of counterfeit drugs. Pharmaceutical items that are fraudulently mislabeled about the source are defined as counterfeit pharmaceuticals [2].Such counterfeit drugs includes medications which leads to mislabeled identity or source for making it as genuine product. These drugs contain no Active Pharmaceutical Ingredient (API) which is defined as incorrect API or repacked expired products. Such drugs might be incorrectly formulated and packed in substandard manner. Health Research Funding Organization has reported that 30 % of the counterfeit are from developed countries. Also, Word Health Organization (WHO) has reported that counterfeit drugs are most important reasons for deaths in developing countries. Counterfeit drugs have estimated the US annual economic loss as $200 billion. Hence an API supplier plays a vital role in distributing the raw materials for drug manufacturer on approval from regulatory agency namely US Food and Drug Administration (US FDA). For instance, US Drug Supply Chain Security Act (DSCSA) has insisted the importance in developing an electronic and interoperable system for tracking prescription drugs across United States for tracking the medications [3]. Some of the countries like China has insisted the needs for the tracking of individuals in supply chain whenever pharma items are stored in Internet Technology (IT) and inventory transaction [4]. However, the implementation of tracking system faces various difficulties in establishing the standards. Current analysis has reported that the pharmaceutical businesses are the first and foremost to engage the industrial market alliance. The advent of internet technology has given rise to a revolutionary development known as blockchain, Consequently, numerous healthcare enterprises have embraced blockchain technology to achieve decentralization, transparency, anonymity and enhanced interoperability [5].

Blockchain has initiated a new application development model which is primarily using the concept of linked list. It includes all the nodes present in the network and it maintains a local copy in all blocks which starts from genesis block. Each of the data segments are linked together in a chain using the hash. Hash functions are one-way; thus, they cannot be changed [6]. Cryptographic structure makes a difficult task on tampering of data and leads to rehashing of all blocks. The characteristics of different types of blockchain are as shown in Table 1. However, blockchain technology is not without its downsides. Regardless of the advantages of blockchain technology, its expansion and use in healthcare applications have created significant research challenges that require further exploration.

**Table 1:** Comparison of the characteristics of three types of blockchains.

Type of Blockchain	Participants	Access	Centrality	Incentives	Consensus
Public chain	All	Free	Decentralization	Needed	PoW/PoS/DPoS
Private Chain	Custom Members	Individual or Intra	Centralization in Multi-level	Selected	PBFT/Raft
Federated chain	Members of Consortium	Members of Consortium	Polycentralization	Selected	PBFT/Raft

**Fig. 1:**
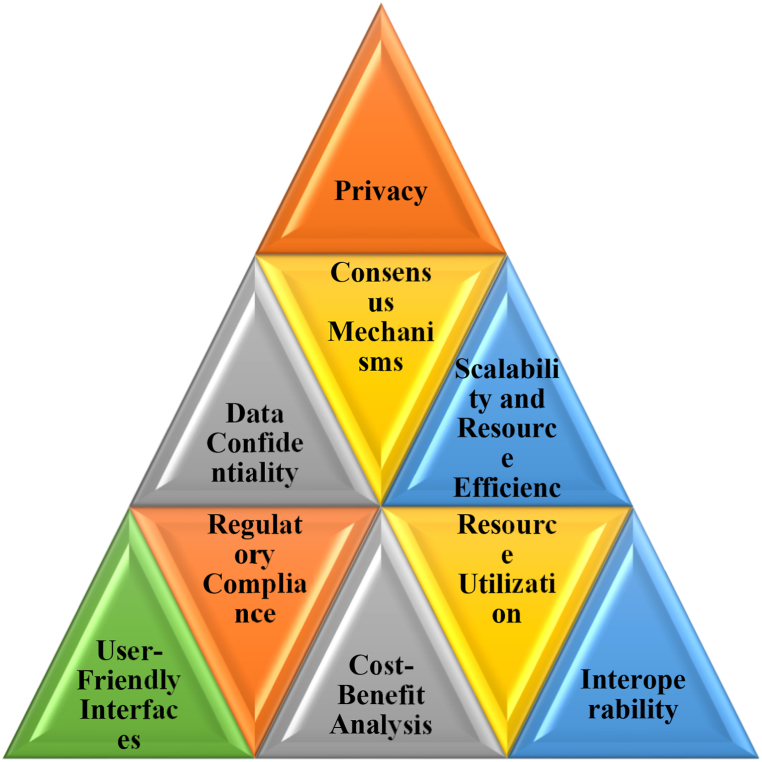
Research Gaps in implementation of Supply chain.

This paper presents a comparative analysis on secure drug supply chain management based on, Hyperledger Fabric and Hyperledger Sawtooth from manufacturing to production stage. Our proposed Trackchain concepts deals with three independent modules namely, initialization of logs using cryptographic structures, smart contracts for automation of records and query service module for retrieving the data. Our first step involves the identification and engagement of stakeholders within the supply chain namely manufacturers, transporters, distributors, hospitals and patients. Out of these stakeholders, we have considered the manufacturer attributes for the implementation and interaction among the stakeholders. Smart contracts are used for the betterment of interaction among stakeholders without human-intervention. Two distinct categories are established namely, selecting the holder by creating the pubic key and creating the medicine record. Additionally, querying about the medicine record is included for the stakeholder interaction. Finally, resource utilization is analysed for all the frameworks in order to determine the effectiveness. Research gaps that requires further exploration in the field of supply chain using Blockchain are as shown in Fig. 1.

As a consequence, the following contributions are made:.

1. We have developed a novel architecture design for handling Pharmaceutical Supply Chain based on Hyperledger Fabric and Sawtooth that guarantees security and accessibility
2. We have added Transfer Chain concepts in Hyperledger Sawtooth which helps in handling assets in an efficient manner
3. We have created a GUI for all the two frameworks for entering the details of the owner and other participants
4. We have compared the resource utilization of the two proposed frameworks.

When supply chain concept is implemented using blockchain, there arises research questions as follows.

1. Which of the framework among Hyperledger Fabric and Sawtooth is suitable for supply chain implementation?
2. How the number of transactions can be increased?
3. How the transactions are handled in Fabric and Sawtooth?

The organisation of paper is as follows: Section 2 discuss about the related works in supply chain. Section 2.1 explains about the key challenges in existing supply chain. Section 3 discuss about the Methodology of TrackChain in Hyperledger Sawtooth. Result Analysis of Hyperledger Sawtooth is depicted in Section 4. Section 5 discuss about the proposed architecture of TrackChain in Hyperledger Fabric. Section 6 discuss the result analysis and comparative analysis.

## 2 Related work

The following Table 2 presents the bibliographic analysis of related works in the field of Supply Chain.

**Table 2:** Bibliographic analysis of Hyperledger sawtooth and supply chain.

Keywords			Published Papers
Supply Chain + Sawtooth			760
Supply Chain + Sawtooth – Review Articles			105
Pharma Supply Chain + Sawtooth Review Articles			21
Pharma Supply Chain + Sawtooth Implementation			77

**Survey on Supply Chain + Sawtooth**			
**Authors**	**Paper Title**	**Implementation**	**Finding**
Mohit et al. [7],	Design and implementation of blockchain-based supply chain framework with improved traceability, privacy, and ownership	Yes	Privacy
Ikram Hasan et al. [8]	Integrated Agri-Food Supply Chain Model: An Application of IoT and Blockchain	No	Conceptual Model
Manaswini Piduguralla et al. [9]	An Efficient Framework for Execution of Smart Contracts in Hyperledger Sawtooth	Yes	DAG parallel scheduler combined with validator of Sawtooth
Tejaswi Kanna et al. [10]	FruitBlock: a layered approach to implement blockchain-based traceability system for agri-supply chain	Yes	Agri Supply-Chain
Abeer Mirdad et al. [11]	A systematic literature review on pharmaceutical supply chain: research gaps and future opportunities	No	Survey Article
Marco Fiore et al. [12],	Blockchain-based Food Traceability System for Apulian Marketplace: Enhancing Transparency and Accountability in the Food Supply Chain	No	Cost Analysis
Anandika Sharma et al. [13]	Blockchain enabled food supply chain management: A systematic literature review and bibliometric analysis	No	Survey Article
Deepthi Sharma et al. [14]	Review Based Analysis with Applications of Blockchain in Food Supply Chain management	No	Survey Article

In recent years, supply chain has gained significant attention due to various reasons such as globalization, technology advancements, supply chain disruptions due to COVID-19, cost pressures and consumer demands. Industries like IBM, Walmart, Maersk, Provenance and VeChain has initiated the supply chain projects integrated with blockchain concepts. Komal Rahul Pardeshi [15] has presented the comparative study of blockchain technology in supply chain management. Supply chain management systems are used in the ideal scenario to determine a product's source and its transformation process, which involves collaboration across several sectors. By ensuring consumer protection, fostering trust, and raising service quality, this promotes industrial efficiency. Health Information Technology (HIT) plays a vital role in enhancing clinical data by outsourcing the data repositories with the help of cloud.

Goodarzian et al. [16] have designed a multi-objective, multi-level, multi-product, and multi-period problem for a sustainable medical supply chain network.. Khan et al. [17], have identified the significant impacts of COVID-19 on supply chain which helps to improve the policies. Niels Hackius et al. [18] has evaluated many use cases regarding blockchain in a positive manner and it also provides various insights over IoT applications with Blockchain. Yue et al. [19] pointed out the fundamental technologies of the blockchain such as encryption, hashing, hash function pointers, digital signatures, binomial trees, and peer-to-peer network propagation. Consensus-building techniques like Proof of Work (PoW), Proof of Stake (PoS) were highlighted.

Gao et al. [20] showed that the majority of currently available blockchain-based traceability systems lack credibility. A supply chain can be termed as a set of three or more entities (organizations or individuals) directly involved in the upstream and downstream flows of products, services, finances, and/or information from a source to a customer. Stefano Bistarelli et al. [21] has designed a framework called *-chain for automating the supply chain deployment to trace and test their own products. Even the systems built on blockchain with advantages for consistency and transparency cannot ensure right traceability for clients without the downstream and upstream trading data of the supply chain.

Abideen et al. [22] stated that in warehouses, blockages and wastes are common problems, especially in the pharmaceutical business with its high stock levels. To prevent the buildup of supply chain lead times, a pharmaceutical warehouse supply chain must have backlog-free, optimal information and material flow. As technology-based solutions are being developed, P. Sanidane et al. [23] recognized that the supply chain of pharmaceuticals requires significant attention because there is a great need for surveillance in the production and transportation of fake, substandard, counterfeit, and grey market medicines, which are responsible for hundreds of deaths each year worldwide.

Wahana et al. [24] met with a problem that often occurs in medical supply chain management, which is the distribution of medications that do not meet the requirements of the local drug inventories. As a result, there is a chance that the medication stock will be reduced at the required location while there is an over-abundance of medication stock at the unneeded location. Manufacturing firms and nations suffer significant financial losses as a result of the supply chain for counterfeit medications. Before a drug reaches the consumer, ownership passes from the manufacturer to the distributor, then to the chemist. The use of their medicine is unknown to the manufacturers. At the exact same time, customers are unaware of the drug's legitimate source. So, blockchain that is permissioned, meaning that only reliable parties are able to join the network and add transactions to it. Survey on existing supply chain is detailed in Table 3.

**Table 3:** Survey on existing supply chain.

Traditional Supply Chain			
Authors	Focus	Type	Finding
Olsen et al. [25]	Food Supply Chain	Survey on Traceable Resource Unit (TRU)	More attributes added on finding the particular product
Abdesselam Bougdira et al. [26]	Traceability and Interoperability	Intelligent traceability in the canneries industry	General Interoperable Traceability Framework
GS1 Data Matrix [27]	Patient Authenticity	Data Matrix for each drug	Transparency is increased
Supriya and Djearamane [28]	RFID based Cloud Supply Chain	Centralised Database	Minimal Attacks

**Blockchain based Supply Chain**			
***Authors***	***Focus***	***Type***	***Finding***
Huang et al. [29]	DrugLedger	Expanded UTXO	Privacy and Authenticity
Faisal et al. [30]	Drug Traceability	Hyperledger	Minimised resources
Muniandy et al. [31]	Anti-Counterfeit Drugs	Ethereum	Transparency
Farouk et al. [32]	BlockRx	Survey on Blockchain and IoT	Finished Goods Traceability
MediLedger [33]	Transfer of Ownership	Legal Transfer of Ownership	Track the medicines ownership
Radanovic et al. [34]	BlockVerify Integration with NFC Embedded Tags	Sensors and Identification Tags	Traceability
Raj et al. [35]	Anti-Counterfeit drugs	Legal Transfer of Ownership	Traceability
Malik et al. [36]	TrustChain	IoT Supported Supply Chain	Minimised Latency
Musamih et al. [37]	Drug Traceability	Ethereum	Traceability
Proposed Work-TrackChain	Drug Tracking	Fabric and Sawtooth	Depicting the Resource Utilization

Wang et al. [38] inferred that when performance of Ethereum, Fabric and Sawtooth is compared, quantity of transactions per second in Ethereum is slower than the latter. In addition to that, it is also concluded that latency of Sawtooth is the lowest among the three frameworks. It was also noted that the entire architecture of Sawtooth is vivid and highly modular and therefore has the capability to be customised more. Saraf et al. [39], has compared all the existing frameworks of Hyperledger and Corda.

Ampel et al. [40] inferred through testing, the maximum throughput is around 2300 tx/sec is achieved. This is far superior to any permissionless blockchain that has been explored, as well as the Hyperledger Fabric results. Sawtooth platform offers two blockchain variations, depending on the need. With the help of this platform, users can create both permissioned and permissionless access. On other platforms, it is possible to offer only one access for the deployment. Bandhu et al. [41], has proposed the system for tracking goods histories (medicine) where the average gas cost for all accounts is 18,027.2, and then the results of the proposed system are compared to state-of-the-art methods. This proposed system highlights a seamless flow of medicines using blockchain and smart contracts. Shasank Kumar et al. [42] has surveyed about supply chain and described the positive relationship between product, supply, demand, and social behavior in IoT adoption.

As an overall inference, it was concluded that supply chain concepts were initated in all frameworks. Resource utilization of the supply chain is not much considered so far. Hence, resource utilization was framed as a specific objective on implementing in two frameworks namely, Hyperledger Fabric and Sawtooth. The increased scalability and ability to run parallel transactions are highlighted as a vital feature in supply chain management. Hyperledger Sawtooth has higher scalability and immutability that can be used to create a pharmaceutical supply chain with scope for cross-chain integration, which makes all information accessible to everyone while maintaining its security and transparency. One of the crucial features of the Sawtooth is parallel transaction verification. This feature ensures the parallel verification of various transactions at the same time. It helps in lessening the workload on the network and thus minimizing the processing time. Interoperability in the pharmaceutical supply chain is crucial for ensuring the efficient and safe flow of pharmaceutical products from manufacturers to patients.

### 2.1 Key challenges of current system

**Fig. 2:**
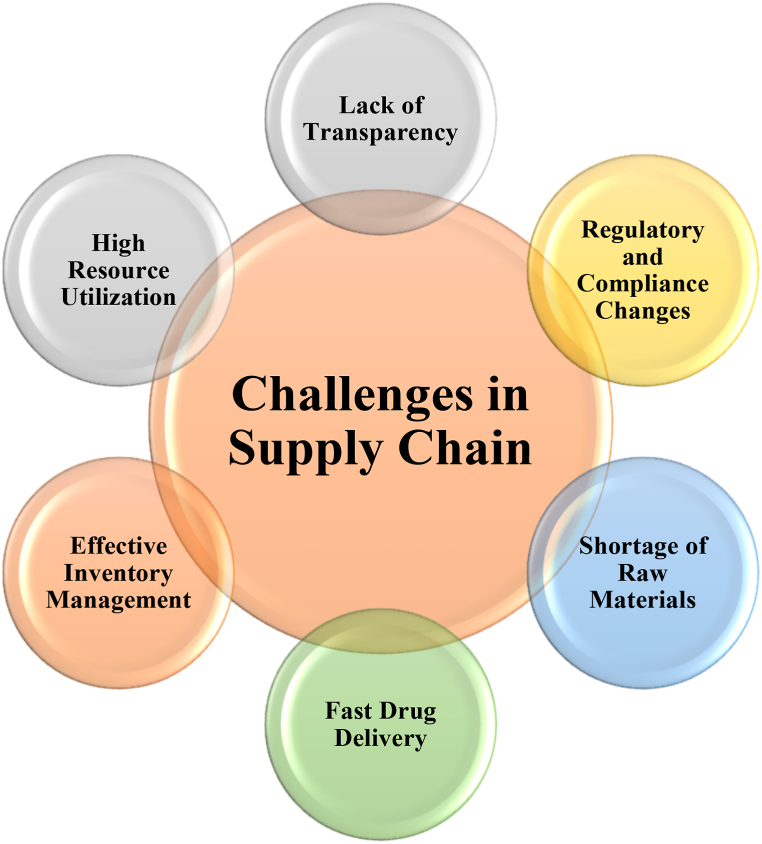
Challenges in supply chain.

During the pandemic situations, stakeholders involved in supply chain were increased day-by-day. Hence, pharma supply chain faces so many challenges. Some of the challenges faced in pharma supply chain are as shown in Fig. 2.

Nonetheless, power usage and scalability pose ongoing challenges across all facets of blockchain technology. Resource optimization is gaining prominence as a focal point of research. As many frameworks have been developed in blockchain, there arises a question on implementation perspective for resource optimization. Hence, the proposed work TrackChain is implemented in two frameworks namely, Hyperledger Fabric and Sawtooth.

## 3 Methodology of TrackChain in Hyperledger Sawtooth

**Fig. 3:**
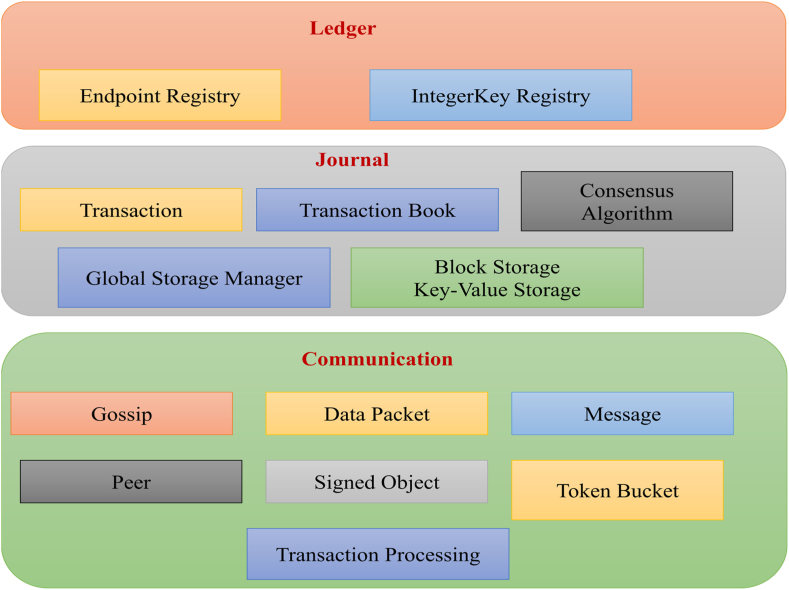
General design of hyperledger sawtooth.

The general design of HyperledgerSawtooth includes three components namely ledger layer, log layer and communication layer as shown in Fig. 3.

The ledger layer consists of Endpoint Registry and IntegerKey Registry as built-in functions. The log layer consists of Transaction, Transaction Block, Consensus Algorithm, Global Storage Manager, Block Storage Key-value Storage which performs the core functions of Sawtooth. Communication layer performs the communication through gossip protocol. Exchange of information is implemented in different types such as transaction block messages, transaction blocks through chat protocol. Token Bucker mechanism controls the transmission speed of data packets.

Our primary objective on TrackChain was implemented using Hyperledger Sawtooth architecture, which offers authentication and security using public key. Sawtooth plug-in components can be easily constructed and then in terms of validator node and transaction processor where manufacturers are added as stakeholders. Transaction Processor was used as an interface with Sawtooth validator and to handle validations of transactions. A simple browser-based client was connected to manage public and private key pairs to submit transactions to Sawtooth REST API. Transfer Chain concept is developed where the medicine assets are created and transferred between the owners designated by public key. For result analysis, data is generated in.json format for single product and it is stored in shared ledger Lightning Memory Mapped Database Manager (LMDB). SHA-256 algorithm is used for producing hash value of each data. For each manufacturer's data, public key is created and then each medicine attributes are added. Hash values are thus verified by decrypting using public key. The proposed architecture of TrackChain is as shown in Fig. 3.

Practical Byzantine Fault Tolerance provides Byzantine fault tolerance for network membership. It has the components as follows.

• Network using PBFT consensus will be able to work if it has atleast four nodes. If nodes are lesser than four, existing network fails.
• PBFT works using fully peered nodes. Static peering is recommended.
• Each node connected to the network install consensus engine using Sawtooth-PBFT-Engine and execute the PBFT consensus engine.
• Each of the nodes run Settings transaction processor for handling PBFT and Sawtooth on-chain settings.
Genesis block specifies the PBFT consensus engine name and version using on-chain settings using sawtooth.consensus.name and sawtooth.consensus.version

### 3.1 Technical architecture of TrackChain

The proposed system contains two Transaction Processors namely Transfer Chain and Cross Chain. Transfer Chain handles transactions related to the medicines in the supply chain and Cross Chain handles interoperability transactions. A GUI is provided where the users can access the sawtooth chain to fetch and add data from the ledger. A pharmaceutical supply chain involving a distributor, manufacturer, pharmacy, and administrator makes all information accessible to everyone while maintaining its security and transparency. The transfer chain is used to perform all the required functions within a particular blockchain. Atomicity is the underlying notion of cross-chain technology. It allows for proper consistency among numerous connected blockchains. It makes it possible to distribute networks among different platforms. Although they are still in the development stage, cross-chain solutions have the potential to significantly speed up blockchain interoperability. It has a great deal of potential to become a workable mechanism in the future to promote improved interoperability solutions.

### 3.2 Components of proposed TrackChain system

**Fig. 4:**
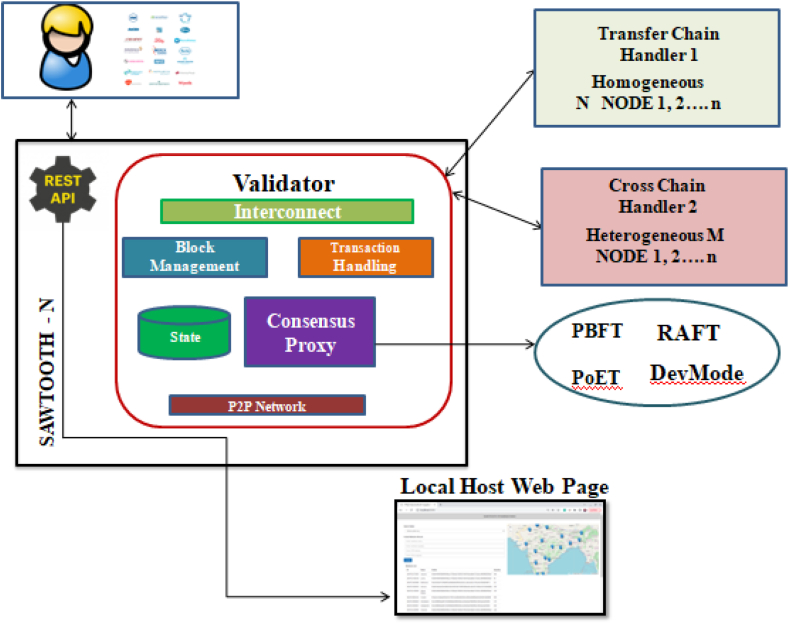
Proposed TrackChain architecture Diagram.

The architecture of the proposed system is given in Fig. 4. The contents of the sawtooth node (i.e the validator, the respective handlers and transaction processors for transfer chain and cross chain), the consensus engine (Devmode consensus) and the REST API creation help the clients interact with the sawtooth chain.

(i) Clients

Clients in the proposed TrackChain include user and manufacturers. Asset Transfer Interoperability issue is majorly focused in the proposed system When manufacturers collaborate for specific product and uses different blockchain framework, interoperability problem occurs. Also, when there is a need to transfer the assets to homogeneous non-connected chain, interoperability occurs. These two major issues are addressed using the transfer chain and cross chain techniques. Clients of Hyperledger Sawtooth have access to a practical REST API that enables them to communicate with a validator in accordance with widely used HTTP and JSON protocols. Once it's up and running, the common language-neutral interface can be used to submit transactions and read blocks.

(ii) Validator

Sawtooth Validator is the core component for running a Sawtooth Distributed Ledger. It orchestrates transaction processing, communication among peers within a network, and consensus between peers, regarding the outcome of the transactions. Once the block is ready,it's broadcasted to other nodes in the network. When a validator receives the block, it validates all the transactions in the block. It is noted that the proposed output states should match the evaluation, otherwise the arrived block will be discarded. If the output state transition is dependent on arbitrary input, there's no deterministic behavior of whether the block is accepted by other validators. Major components of validator are as follows.

a) Transaction Processor

Sawtooth consists of Transaction Processor which is equivalent to smart contracts or chain codes in a traditional blockchain system. Modifications to the state of the blocks in the chain are performed by creating transactions. Any Transaction Processor written should be deterministic in nature. Deterministic behavior is that for a given input state, the logic in the smart-contract should always evaluate to a specific output state. For validation, the transactions are sent to the transaction processor. A transaction processor reads the data from the global state (i.e data stored until current chain head), applies what it needs to do and computes the output state (i.e data stored on the ledger, if current block is accepted).When a transaction is sent to the validator, it is broadcasted to other nodes in the network and added in a pending queue. In the proposed TrackChain, two transaction processors are connected to the validators, namely Transfer Chain and Cross Chain. Transfer Chain works on transferring the blocks to the other homogeneous chain (i.e) Hyperledger Sawtooth via sidechain concepts. Cross Chain works on transferring the assets to the heterogeneous chain (i.e) where stakeholders uses different framework.

b) Consensus Proxy

Based on the command from the consensus engine, the validator is responsible for building the block. It will take pending transactions, validate them, and put them in a block. Protocol Buffers, which offer a dialect-neutral, platform-neutral, extensible mechanism for serialising structured data in a forward-compatible and backward-compatible manner. This mechanism is used by Sawtooth node to serialise messages used for communication. Validator uses REST API to submit transactions and get results as a "black box." The storage of data in the proposed system is done using Lightning Memory Mapped Database Manager (LMDB). As LMDB uses a shared memory model, all the threads can access the memory simultaneously from a single address space. As a B tree-based database management library, LMDB is much more straightforward. All data queries return data straight from the mapped memory, and the full database is visible in the memory map.

### 3.3 Algorithm for proposed TrackChain

(I) Start Docker
a) sawadmkeygen
b) sawtoothkeygenmy_key//Create a validator key
c) sawset proposal create//A signing key for the user is created.
d) consensus.algorithm.name = Devmode
e) consensus.algorithm.version = 0.1//The type and version of the consensus algorithm are initialised.
f) The network endpoint URL for the validator is set.
g) Service endpoints for validator components are initialised.
h) The consensus engine image is initialised.
1. A REST API is initialised to get input data from clients.
2. Two transaction processors and their respective handlers are created to execute parallel processing.
3. While the REST API is running and the port is open.
i. A transaction processor and handler are created for the medicine details.
ii. Another transaction processor and handler are created for maintaining key-value pairs.
iii. End while and close port.
(iii) Transaction Handling

To add a value to a block, a transaction is required. It has to be wrapped inside a batch and submitted to the block. There may be one or many transactions in a batch. Every transaction in the batch is successfully sent to the block if the batch is successful; otherwise, every transaction in the batch is discarded. Upon receiving the transaction, the receiver can check the transaction using the transaction header. Transaction processing is managed through a local component network. In a batch of one or more transactions, the client transmits a transaction to a validator. Through the REST API, the validator receives the transactions and adds them to the blockchain.The header field is the serialised version of the transaction header. The sender, or the person who inserts data into the blockchain, must fill out a number of fields in the transaction header. The signer's public key is added to signer_pubkey. The serialised header is signed using this key, and the resulting signature is added to the header signature field. The transaction family in the transaction header defines the possible transactions that the current transaction is allowed to perform. Simply, the dependencies field in the components of the current implementation includes containers for a validator to initialise the consensus algorithm to be used and generate its own key and a user key. The component known as the validator is ultimately in charge of validating batches of transactions, aggregating them into blocks, upholding network consensus, and organising communication between clients, other validators, and transaction processors. Transaction processors are used to make on-chain changes during the execution of all the blocks. The REST API is used to connect clients to on-chain activities.

(iii) DevMode Consensus

The validator is used to set up the network and service endpoints for its components. The consensus engine is used to run Devmode consensus. Each consensus type has a consensus engine that communicates with the validator. Each node in the network must run the same consensus engine. The settings transaction processor is used to apply changes to on-chain settings. The transaction processor defines the business logic for the application. The state can be changed by establishing and implementing transactions. A transaction is created by a client and sent to the validator. The validator process the transaction and cause a change in state. Clients can communicate with a validator utilizing widespread HTTP and JSON standards.

### 3.4 Transaction architecture

**Fig. 5:**
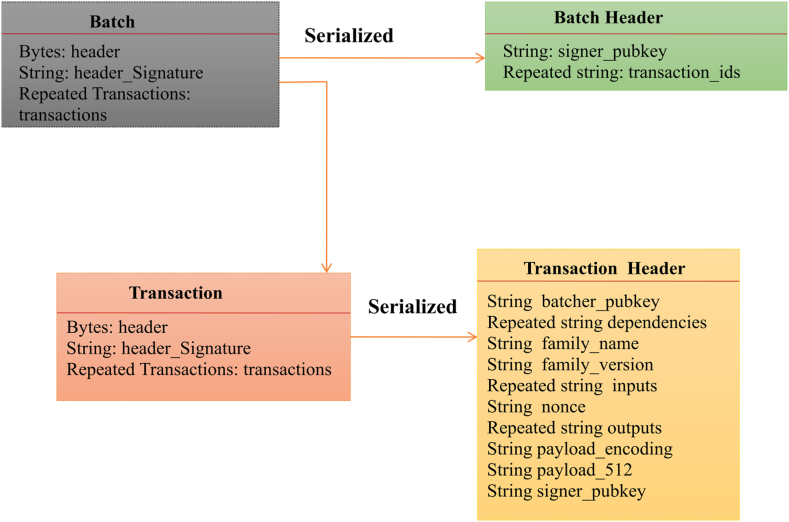
Transaction attributes.

Batches and Transactions in Hyperledger Sawtooth includes Batch, Batch Header, Transaction, and Transaction Header as shown in Fig. 5.

To include an item within a block, a transaction is employed, requiring it to be enclosed within a batch and then submitted to the block. A batch can encompass a single transaction or multiple transactions. In the event of a successful batch, each transaction within it is considered to have been executed successfully. System configuration used for the implementation of Trackchain is tabulated in Table 4.

**Table 4:** Experimental setup.

Parameters	Value
Machine specification	CPU: Intel Core i7, RAM: 8 GB
Hyperledger Fabric Client	Python Client
Centralised database	CouchDB
Operating System	Ubuntu 20.04
Language	Go

The components of the current implementation include containers for a validator to initialise the consensus algorithm to be used and generate its own key and a user key. Validator is the component ultimately responsible for validating batches of transactions, combining them into blocks, maintaining consensus with the network, and coordinating communication between clients, other validators, and transaction processors. Transaction processors are used to make on-chain changes during the execution of all the blocks. REST API is used to connect the clients to on-chain activities.

## 4 Result analysis

**Fig. 6:**
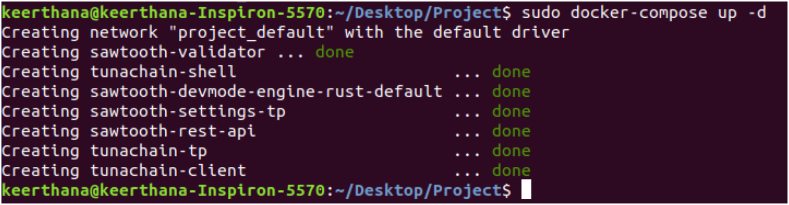
Docker initialization.

Once the docker is up, all the necessary docker containers get initialised. The initialised dockers shown in Fig. 6 which include containers for the validator, the consensus engine for devmode consensus, settings transaction processor, a shell container, REST API, transaction processor and a client container.

**Fig. 7:**
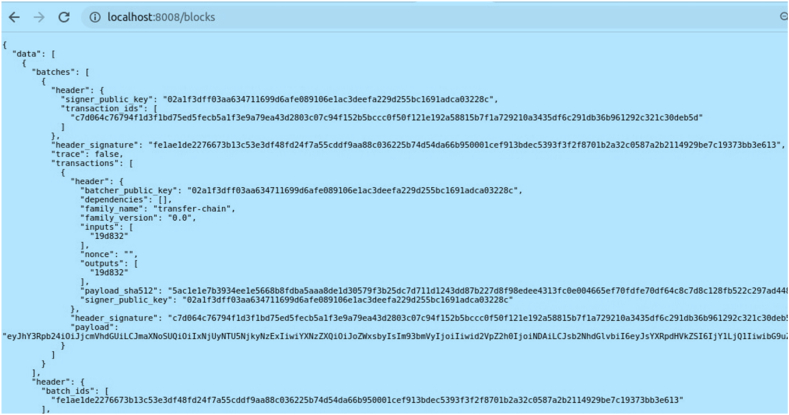
Block metadata.

Blocks which are added into can be viewed as metadata (i.e.), block header, block number, previous block's hash, state root has are displayed as shown in Fig. 7.

**Fig. 8:**
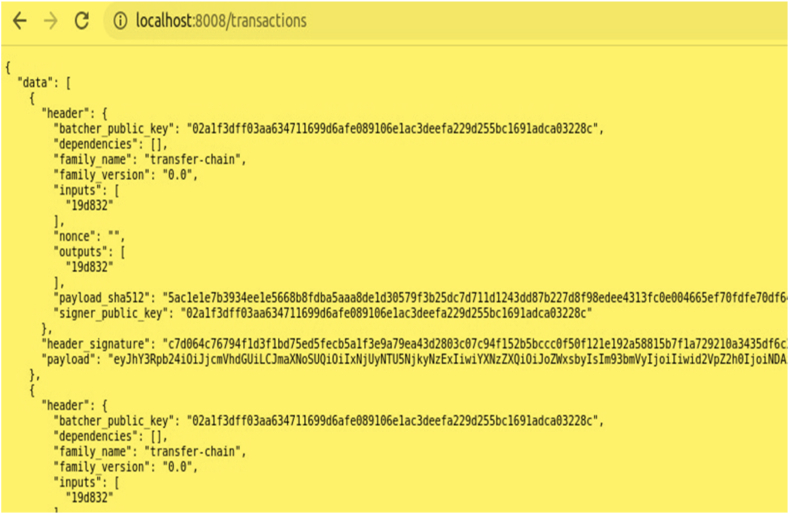
Transaction metadata.

Transactions are arranged from the blocks and the details about the transactions are stored as metadata (i.e.), header, transaction id, header signature, batch's public key, family name, family version are displayed shown in Fig. 8.

**Fig. 9:**
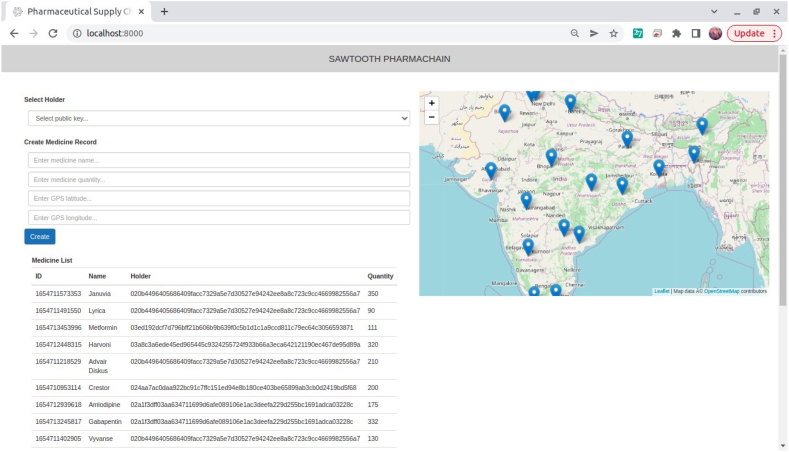
User interface.

User Interface for the stakeholders to create the record in the database is as shown in Fig. 9.

The details about each medicine, the quantity and their holder are displayed as a table in the localhost webpage. Each location of medicine is marked on the map. The collected data provides the location of medicine in a pinned manner.

## 5 Hyperledger Fabric

**Fig. 10:**
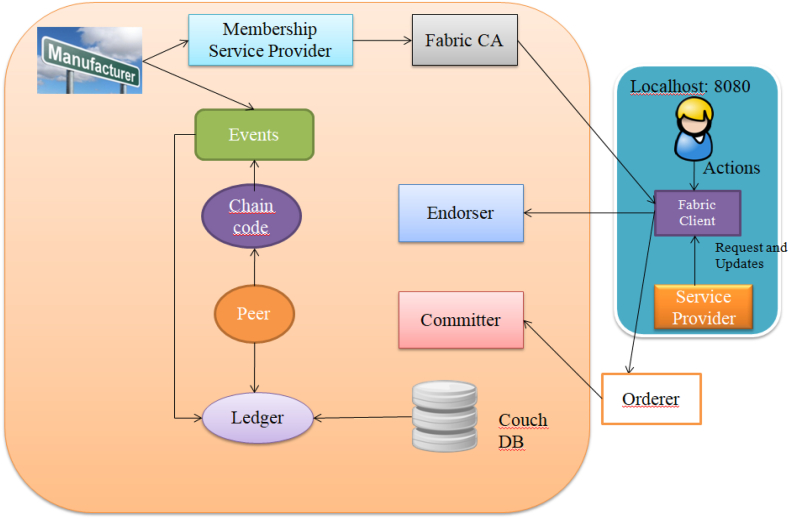
Proposed architecture of supply chain in hyperledger Fabric.

The proposed architecture of supply chain in Hyperledger Fabric is shown in Fig. 10.

### 5.1 Fabric components

Blocks are created by the ordering service, and then validated and committed by peers. An organization in Fabric has to create a Public Key Infrastructure (PKI) which has to contain a Certificate Authority (CA) that will issue digital certificates for the organization identities. Certificate Authorities issue identities by generating a public and private key. These keys form a key-pair that can be used to prove identity. Since a private key can never be shared publicly, a mechanism is required to enable that proof. Therefore, Membership Service Provider(MSP) is used.

#### 5.1.1 Membership Service Provider

Membership Services authenticates, authorizes, and manages identities on a permissioned blockchain network. Authentication scheme is based on PKI abstraction. In Hyperledger Fabric, an organization is identified by its MSP. Everything that interacts with a blockchain network (peers, applications, admins, and orderers) acquires their organizational identity from their digital certificate and their Membership Service Provider (MSP). Each organization is identified by its Membership Service Provider identification (MSP ID). For example, a peer uses its private key to digitally sign, or endorse a transaction. MSP involved in the ordering service contains the peer's public key. It is then used to verify if the signature attached to the transaction is valid or not. The private key is used to produce a signature on a transaction that only the corresponding public key which is part of an MSP can match. Thus, the MSP is the mechanism that allows identity to be trusted and recognized by the rest of the network without ever revealing the member's private key.

#### 5.1.2 Channel

A channel is a private subnet of communication between two or more specific network members which ensures confidentiality. Channels isolate peers and ledger data to provide private and confidential transactions on the blockchain network. Each transaction on the network is executed on a channel. During such transactions, each entity participating must be authenticated and authorized to transact through channel. Each peer that joins a channel, has its own identity given by a membership services provider (MSP), which authenticates each peer to its channel peers and services. Fabric uses Access Control Lists (ACLs) to manage access to resources. Fabric contains a number of default ACLs. The ACL is part of a channel's configuration. Channels can be added to the network and members can use the specified channels. The required peers can also be permitted to join the channel. Each channel includes ordering service nodes, peers, shared ledger. Chaincodes are instantiated on the channel and ACL.

#### 5.1.3 Orderer

Orderers are responsible for managing the roster of approved organizations permitted to initiate channels and sequence transactions within a block. The ordering service establishes a common communication channel accessible to both clients and peers, offering a messaging broadcast function for transaction-containing messages. Importantly, the ordering service operates autonomously from peer processes and prioritizes transactions on a first-come, first-served basis across all network channels. Clients link to the channel and can transmit messages onto it, subsequently disseminated to all peers. Through the orderer services, transactions are "propagated" to orderers and then "distributed" as blocks to the relevant channel. The group of organizations supervised by the orderer is termed as the consortium. The ordering service may comprise a single or multiple orderer nodes. Consensus refers to the alignment on the transaction order among individual ordering service nodes. Hyperledger Fabric offers support for three distinct consensus mechanisms or implementations: SOLO, Kafka, and Raft.

#### 5.1.4 Peers

A Peer functions as a network entity responsible for maintaining a ledger and running chaincode containers, enabling the execution of read and write operations on the ledger. Peers are owned and managed by network members, serving as the foundational components of the network infrastructure. They are tasked with preserving the network's state and holding a replica of the ledger, along with hosting instances of both ledgers and smart contracts. There are two primary roles for peers: endorsing peers and committing peers. Endorsing peers are responsible for simulating and endorsing transactions. They play a crucial role in preventing unstable or non-deterministic transactions from propagating through the network. In contrast, committing peers validate endorsements and verify transaction outcomes before appending these validated transactions to the ledger specific to the channel. This process ensures the integrity of the blockchain.

#### 5.1.5 ChainCode

In Hyperledger Fabric, smart contracts are encapsulated within chaincodes. Chaincode is a program written in Go and Node.js, plays a pivotal role in task implementation and acting as an interface to the Fabric ecosystem. Chaincode is responsible for initializing and managing the ledger state through transactions submitted by applications. To ensure security and isolation from the endorsing peer process, it is imperative that chaincode operates within a secure Docker container.

#### 5.1.6 CouchDB

CouchDB serves as an optional, alternative state database that seamlessly integrates with Fabric. It operates as a key store and can be effortlessly incorporated into the Fabric architecture. One of its significant advantages is its ability to represent ledger data in the form of JSON, enhancing query efficiency, especially when dealing with extensive databases. This feature also empowers users with the ability to execute comprehensive and intricate queries, making it particularly valuable for complex data retrieval needs. CouchDB operates independently as a distinct database process, running alongside the peer. These components are linked to the Fabric client, which is authorized by the Fabric Certificate Authority (CA). The Fabric CA serves as an intermediary between users seeking updates or actions to be executed and the transmission of transactions into the blockchain.

### 5.2 Methodology of TrackChain in Hyperledger Fabric

The framework connectivity between Hyperledger Fabric components are established. Docker containers are created to form the core infrastructure, encompassing the Fabric Certificate Authority, peers, orderers, and CouchDB. Additionally, a Command Line Interface (CLI) is established, facilitating the execution of chaincode within the Fabric environment. Throughout this setup, monitoring is maintained over the creation of each container and the establishment of communication channels by Membership Service Providers (MSPs) and other logging mechanisms among the relevant containers. Utilizing the Certificate Authority (CA), an administrative user is enrolled and generated, accompanied by the provision of an MSP ID and password, as well as the private key and certificate obtained during the enrollment process. As the existence of an administrator is a prerequisite for user creation, a user is subsequently established. Users are registered and enrolled through the same CA that administered the creation of the administrator, and similarly obtain the corresponding set of credentials as the administrator. A static file server is configured, directing it towards the "client" directory. The designated port number is specified and preserved. Server is activated and listened on the designated port, enabling the view of the output on the local host. The implementation details are as follows.

**Fig. 11:**
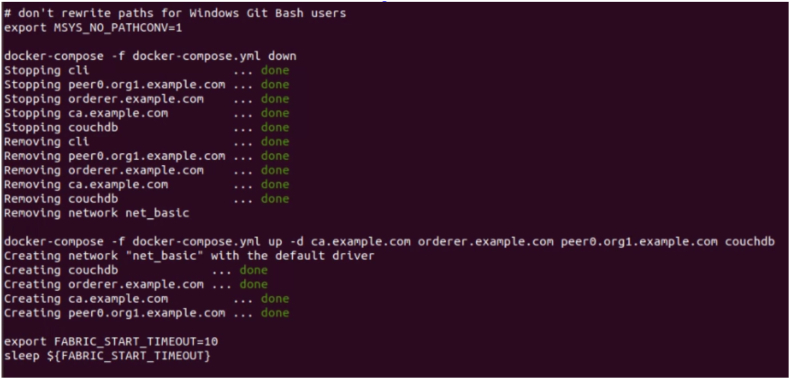
Stop all existing Dockers.

1. Stop and remove all existing dockers to avoid saved outputs. Create the peer, orderer, CA and couchDB containers as shown in Fig. 11.

**Fig. 12:**
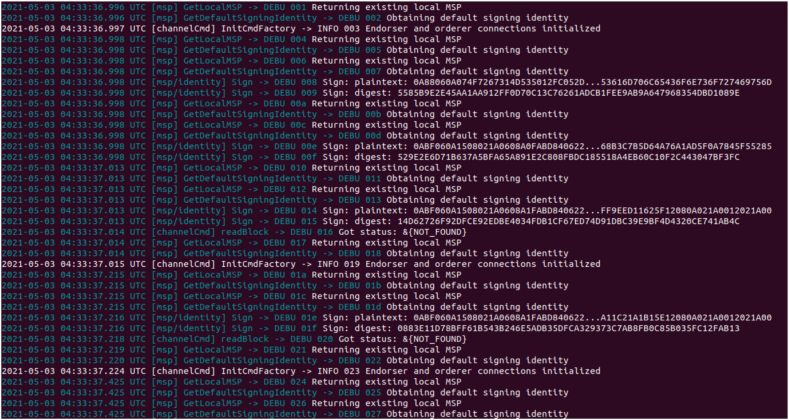
Establishing new channels.

**Fig. 13:**
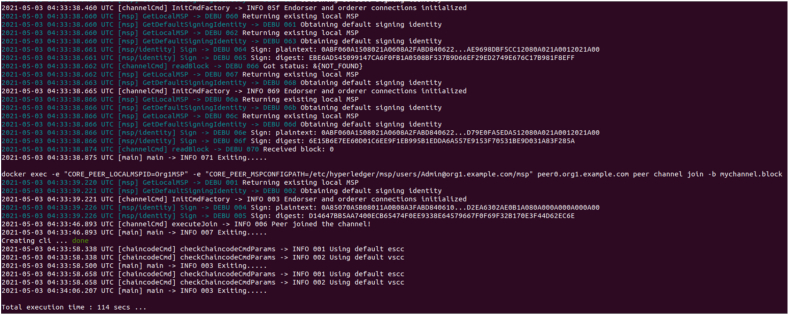
Endorsing channels.

**Fig. 14:**
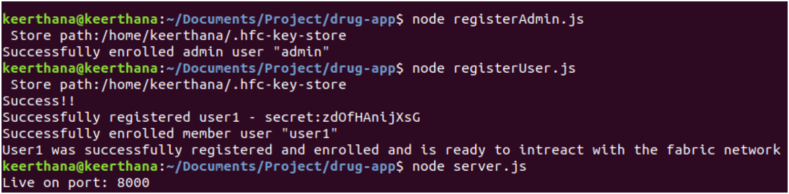
Admin Registration.

2. Channels are created for the peer with the same admin and MSP ID using the genesis block. The channels are joined to the peer using channel.tx and channel.block as shown in Fig. 12.

3. Due to the logging levels set along with other environment variables, MSP logger and ChannelCmd logger can print the status of the signing identities. Validation and endorsement of the channel connections are established. The CLI is created and the chain code is instantiated as shown in Fig. 13.

4. Admin is created and enrolled with the peer. Since an admin exists, a user can be created where they are enrolled to the same peer as shown in Fig. 14. Additionally they are registered where a username and a password/secret is generated along with certificates and private keys for both the admin and the user.

**Fig. 15:**
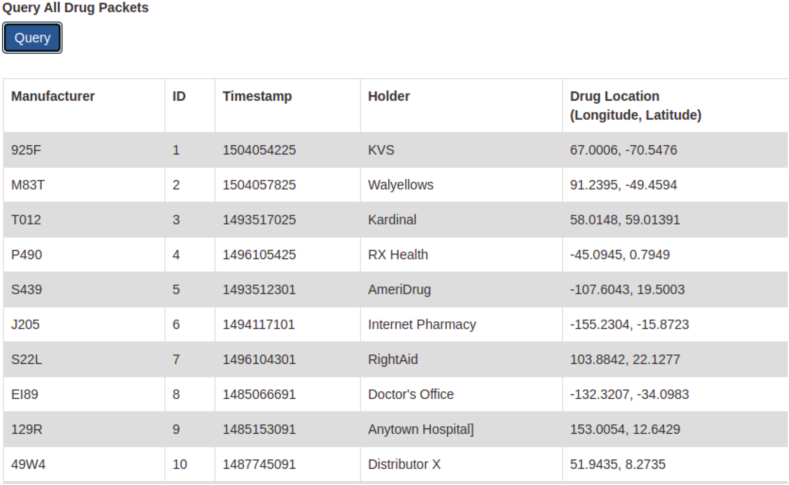
Database of all drugs.

**Fig. 16:**
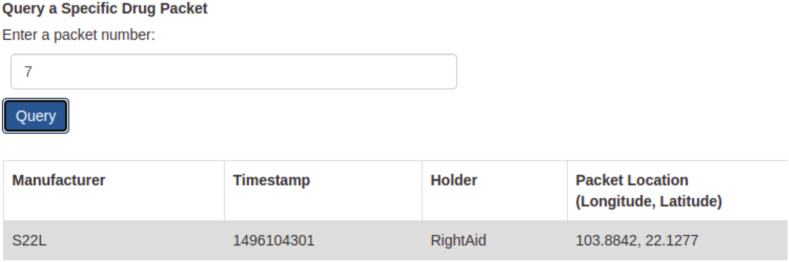
Query on specific Drug.

In localhost:8000 we can see options to Query all drug packets and also a specific drug packet can be queried using its ID as shown in Fig. 15, Fig. 16 respectively.

## 6 Performance analysis

The performance analysis for the proposed TrackChain is shown in Fig. 16. Based on the implementation, we can derive the resource utilization of docker container and find out their individual CPU and Memory usage. From the barchart, it is seen that during execution, the components which perform the transaction activities uses more CPU. For example, the consensus engine, the settings transaction processor and the validator uses more CPU during execution due to their function on the chain. The consensus engine decides which block's transactions have to be executed and at which time. The settings TP needs to handle the sawtooth on chain changes to the transactions. The validator needs to monitor the authenticity of each transaction on the chain. Further, the transfer chain uses more memory as it gets input data from the user which is done using the REST API.

### 6.1 Comparative analysis

**Fig. 17:**
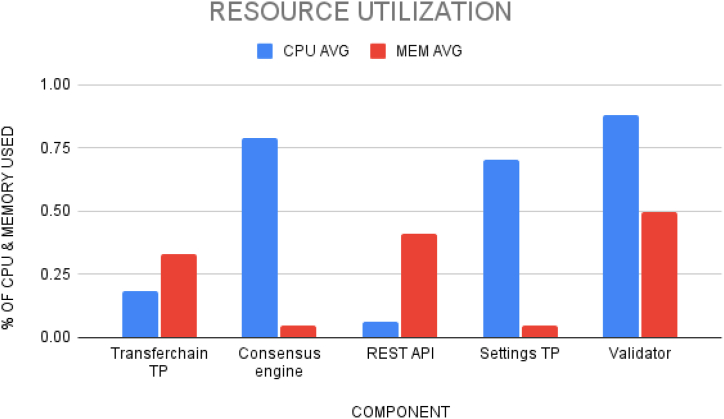
Resource utilization.

Actions and terminologies used in Hyperledger Fabric and Sawtooth are tabulated in Table 5. Fig. 17 shows the difference between CPU usage in each component of both Fabric and Sawtooth applications. The components along the x-axis in order are Peer1 and SettingsTP, Peer2 and Transfer chain TP, Certificate Authority and Validator, Orderer, and Consensus Algorithm.

**Table 5:** Comparison on terminologies in Fabric and sawtooth.

Actions	Hyperledger Fabric	Hyperledger Sawtooth
Commit Transactions	Peers	*Transaction Processors (TP)*
Execute Transactions	Orderer	*Functions*
Validate Transactions	Certificate Authority	*Validator*
Service Provider	Orderer	*Consensus Algorithm*

## 7 Statistical analysis

**Fig. 18:**
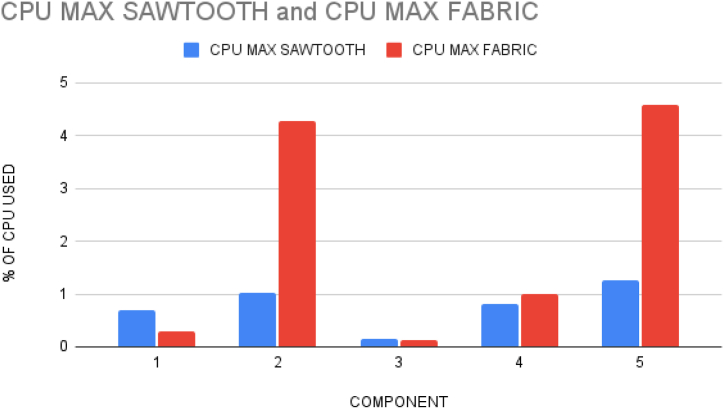
CPU usage Sawtooth vs Fabric.

**Fig. 19:**
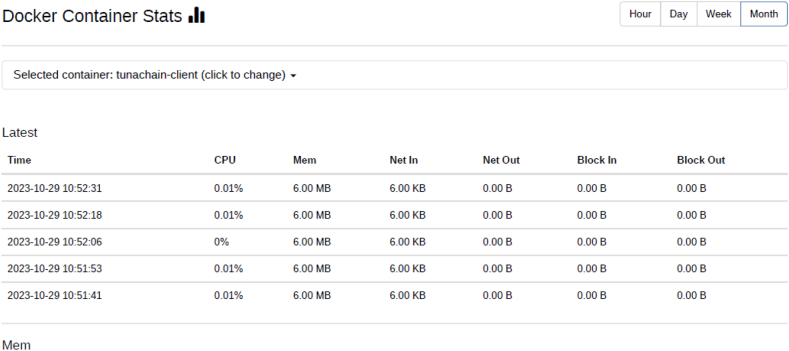
Docker statistic of tunachain-client.

**Fig. 20:**
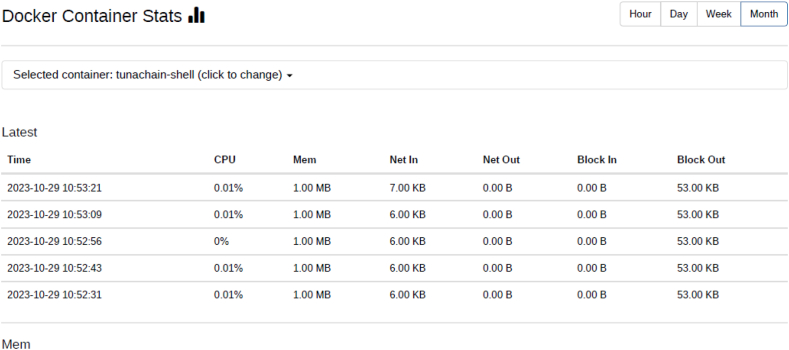
Docker statistic of tunachain-shell.

**Fig. 21:**
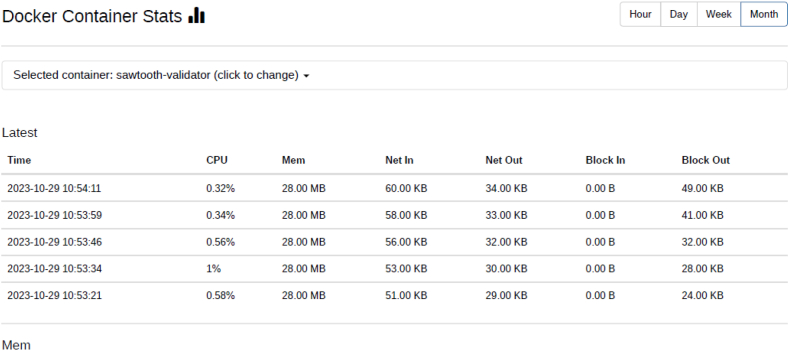
Docker statistic of sawtooth-validator.

**Fig. 22:**
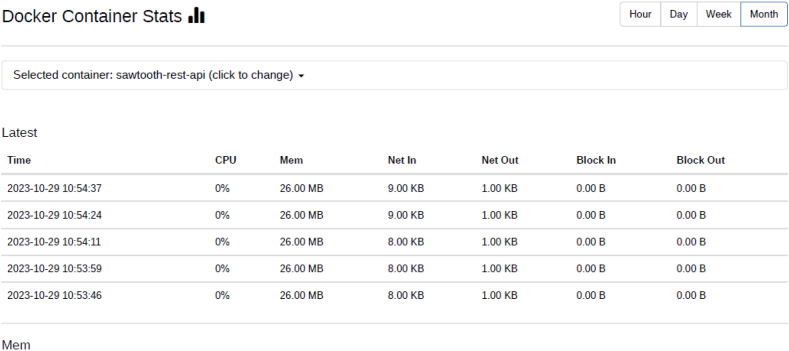
Docker statistic of sawtooth rest API

Docker containers for validator, the consensus engine for devmode consensus, settings transaction processor, a shell container, REST API, transaction processor and a client are initiated. The time duration for the docker compose to up and down were analysed using time commands of docker. Real time provides wall clock-time from initialization to finalize the call. This includes the elapsed time including the time slices used by the processes and the time the process was blocked. User time defines the amount of CPU time spent in user-mode within the process and System time defines the amount of CPU time spent in kernel within the process. Time statistics of docker, the percentage of the host's CPU and memory the container is using are tabulated in Table 6, Table 7 respectively. CPU usage of Hyperledger Sawtooth and Fabric is shown in Fig. 18. Docker container statistics of Tunachain-client, shell, sawtooth-validator and sawtooth-rest-API are shown in Fig. 19, Fig. 20, Fig. 21, Fig. 22 respectively.

**Table 6:** Time statistics of docker.

Category	Real	UserMode	Kernel Mode
Docker initialised for first time	33.87s	0.054s	0.091s
After installation of six containers	2.418s	0.003s	0.006s
When docker shuts down	21.562s	0.001s	0.005s

**Table 7:** Percentage of CPU, memory usage and network utilization in docker.

Category	CPU (%)	Memory (%)	Network Out
Docker in Running state	2.28	*67.882*	*90.06*
Docker in Running down	*0.26*	*30.16*	*15.01*

## 8 Theoretical contributions

Awan et al. [43] has described the theoretical contributions to the big data on manufacturing agility. The proposed work TrackChain has made contributions to the two frameworks namely, Hyperledger Fabric and Sawtooth. The literature papers has focused on two frameworks with various stakeholders and results were interpreted specifically. Resource utilization is not considered as the main objective in the existing literature. But, TrackChain implementation has involved same participants from the existing literature for analysing resource utilisation. Thus, from the proposed work, Hyperledger Sawtooth shows minimal resource consumption on implementation. From the literature survey, it is inferred that the proposed work TrackChain is the first to provide the implementation in two different frameworks and thus provide the resource utilization statistics.

## 9 Conclusion and future work

Pharmaceutical supply chain management has emerged as a central focus in healthcare research. Blockchain technology, renowned for its transparency, has significantly bolstered the effectiveness of supply chain concepts in this domain. Consequently, the TrackChain was executed across two distinct blockchain frameworks: Hyperledger Fabric, and Sawtooth. Thus, proposed Trackchain implementation on Hyperledger Fabric and Sawtooth was discussed in Section 3 and Section 5 respectively. The judicious utilization of resources is of paramount importance in implementing pharmaceutical supply chain concepts. Among the two frameworks examined, Sawtooth exhibited the most efficient resource utilization. Therefore, for a pharmaceutical supply chain concepts when minimal resources on CPU and memory are considered, Hyperledger Sawtooth will be the most preferred framework. Notably, when comparing Hyperledger Sawtooth and Fabric, it was discerned that Fabric demonstrated higher CPU utilization. Within Hyperledger Sawtooth, validators were found to consume more CPU and memory resources. Looking ahead, the implementation of a Cross-Blockchain Connectivity Protocol (CBCP) is a prospective avenue for accommodating an increasing number of stakeholders utilizing various blockchain frameworks. Given the variability of supply chain stakeholders across different companies, expanding the TrackChain project to encompass additional stakeholders is a plausible extension. Furthermore, future work may involve the development of interoperability mechanisms between these frameworks using cross-chain technology. Additionally, the study did not include a comparative analysis of resource usage, specifically regarding RAM, storage, and database capacity. These omissions could be considered as constraints in the proposed research, and potential avenues for enhancing resource optimization might be explored in future endeavors.

## CRediT authorship contribution statement

**NagaSudha C.M:** Writing – review & editing, Writing – original draft, Visualization, Software, Project administration, Methodology, Conceptualization. **Jesu Vedha Nayahi J:** Writing – review & editing, Validation, Supervision, Project administration, Investigation.

## Data availability statement

Data related to this study has not been deposited in a publicly available repository, but will be available upon request.

## Additional information

No additional information is available for this paper.

## Declaration of competing interest

The authors declare that they have no known competing financial interests or personal relationships that could have appeared to influence the work reported in this paper.
